# A Long-Term Survivor of Trisomy 18

**DOI:** 10.7759/cureus.51491

**Published:** 2024-01-01

**Authors:** Anusha Garg, Trudy C Wu

**Affiliations:** 1 Biology, Troy High School, Los Angeles, USA; 2 Radiation Oncology, University of California Los Angeles, Los Angeles, USA

**Keywords:** trisomy 18 syndrome, quality-of-life, genetics, pediatrics, trisomy 18

## Abstract

Trisomy 18 is known for its severe prognosis, with most affected infants not surviving beyond a week, but this report sheds light on a remarkable case of a two-and-a-half-year-old girl born with Trisomy 18 who has thrived due to specialized medical care. Despite a complex medical profile, including congenital heart defects and hepatoblastoma, this patient underwent successful treatments, including multiple surgeries and chemotherapy. This case report showcases how modern medical advancements and multidisciplinary care can defy the historically grim prognosis associated with Trisomy 18, providing hope for improved outcomes and a better quality of life (QOL) for affected individuals and their families.

## Introduction

Behind Down syndrome (Trisomy 21), Edward’s syndrome (Trisomy 18) is the second most common trisomy due to meiotic nondisjunction. Common findings on a prenatal ultrasound include intrauterine growth restriction, polyhydramnios, abnormal fetal hand positioning, and congenital heart and kidney defects [1]. More female infants are diagnosed with this disease and have a lower mortality rate, although very few survivors live beyond a week, let alone five years [1]. Prominent phenotypic features include micrognathia and small mouth, deformed ears, webbed toes, overlapping fingers, and short stature [2]. Clinically, Trisomy 18 impacts a wide range of organ systems [1,3-4]. In about half of infants, congenital heart disease may be diagnosed, most commonly, as a ventricular septal defect (VSD) or patent ductus arteriosus (PDA). In the central nervous system (CNS), cerebellar hypoplasia, hydrocephalus, and neural tube defects may be present, manifesting as seizure disorder and/or deficits in basic motor skills. With respect to genitourinary (GU) and gastrointestinal (GI) findings, infants commonly are born with a horseshoe kidney or Meckel’s diverticulum, respectively. In a small subset of patients, cancer may develop, including hepatoblastoma, Wilms Tumor, or aortic valve tumors. Finally, skeletal development issues are common resulting in a high probability of scoliosis [5].

Around 50% of patients diagnosed with Trisomy 18 in utero are born stillborn. Of those who survive, most will pass away within the first six months of life. In the subset of patients who receive medical treatment, about 33% will live to over one year and 10% to eight years [6]. Medical interventions such as cardiac surgeries, tracheostomy, and other palliative measures are critical to prolong survival. Herein, we present a case of a two-and-a-half-year-old girl who was born with Trisomy 18 and is currently in good functional health after receiving tertiary-level medical care. 

## Case presentation

**Figure 1:**
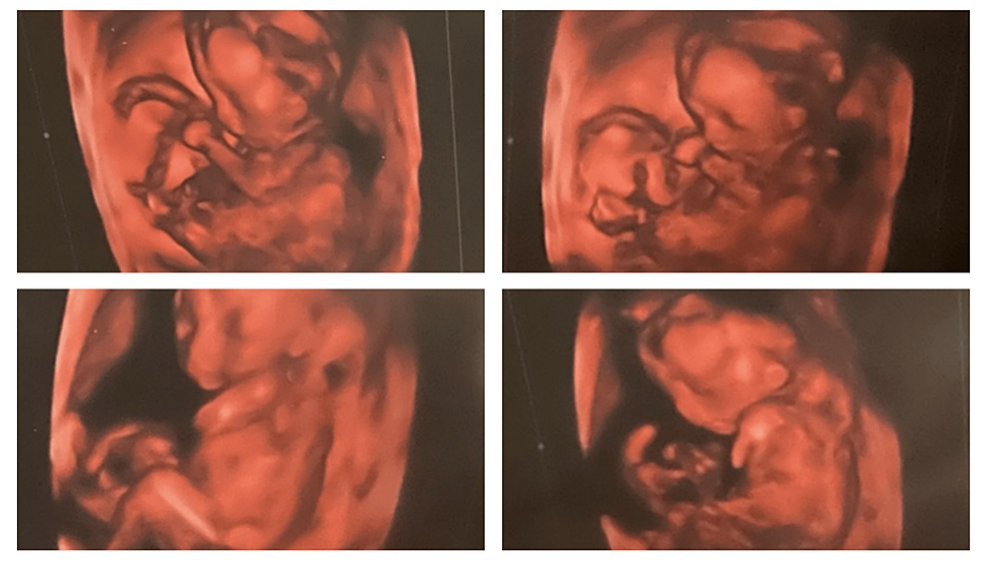
Prenatal Ultrasound

The patient is a two-and-a-half-year-old female who was born with Trisomy 18 in the spring of 2021. On routine noninvasive prenatal testing, the infant screened positive for Trisomy 18. This was confirmed by amniocentesis and subsequent anatomy scan. Prenatal ultrasound at six months again demonstrated sonographic findings of Trisomy 18 (Figure 1; Table 1). The patient's karyotype was full Trisomy 18. 

**Table 1:** Common Malformations Associated With Trisomy 18 and Treatment Options PDA, patent ductus arteriosus

Malformation	Incidence	Treatment Option
Septal defect	>75%	Septal repair
PDA	>75%	PDA ligation
Horseshoe kidney	25-75%	
Tracheoesophageal fistula, esophageal atresia	5-25%	Gastrostomy +/- tracheoesophageal fistula ligation +/- primary esophageal atresia repair
Meckel diverticulum	5-25%	Excision of the Meckel diverticulum + intestinal repair
Cerebellar hypoplasia	5-25%	
Agenesis of corpus callosum	5-25%	
Orofacial clefs	5-25%	Surgical repair

In the setting of a poor and often fatal prognosis, termination of the pregnancy was recommended, and life-altering surgeries post-birth were not offered at several large regional tertiary medical centers. This prompted the patient’s family to seek additional expert consultation at other medical centers including The University of Cincinnati Children’s Hospital. 

At birth, the patient was noted to have agenesis of the corpus callosum with volume matter loss, cerebellar and vermian hypoplasia, high-grade vesicoureteral reflux, and tracheobronchomalacia. Upon echocardiogram, she also had multiple congenital cardiac deformities including a perimembranous VSD with inlet extension, patent foramen ovale (PFO), PDA, polyvalvular dysplasia, and fenestrated atrial septal defect (ASD). Early on in her infancy, the patient developed worsening heart failure prompting VSD repair, ASD closure, and PDA ligation at four months and 17 days of age. At the same time, she underwent permanent tracheostomy placement. 

About two months following the patient’s cardiac surgeries, a liver mass was incidentally detected upon imaging along with high levels of alpha-fetoprotein. The patient subsequently underwent a laparoscopic left hepatectomy and lobe resection for multifocal hepatoblastoma. Post-operative MRI imaging was negative for residual disease. She then received six cycles of adjuvant cisplatin. Following a prolonged hospitalization, the patient was discharged to return home. At the time of this writing, the patient has avoided hospitalization for the past 18 months and currently resides with her parents and sibling. To date, she has severe verbal and motor developmental delays along with hypotonia and severe bilateral hearing loss. 

## Discussion

Trisomy 18 is a devastating congenital chromosomal disorder that affects numerous organ systems, leading to distinctive characteristics and life-threatening conditions. Initially identified in 1960, the prognosis for individuals with Trisomy 18 has historically been poor, with infants seldom surviving beyond their first year of life despite medical intervention. In this report, we present a case of a patient with Trisomy 18 who, following intensive medical treatments, has survived to almost two years of age with a favorable level of functioning. 

Cardiac defects are one of the most prevalent anomalies, present in 90% of patients with Trisomy 18 [7]. Our patient was born with multiple cardiac malformations including a perimembranous VSD and PDA for which she underwent VSD repair and PDA ligation at four months and 17 days of age. In addition to the complexity of cardiac anomalies requiring an expert and multidisciplinary team at a select large tertiary academic centers, access to care can also be challenged by financial, emotional, and logistical factors, oftentimes prompting a significant travel burden to seek medical care [8,9]. Despite these hardships, studies have shown that offering cardiac surgery dramatically improves overall survival at 12 months to 25%, compared to those receiving conservative treatment at 9% (HR 5.53, p=0.006) [10]. In addition to improved survival, evidence shows that infants experience a better quality of life (QOL) following cardiac surgery, both physically and emotionally. Although QOL may be difficult to objectively measure for infants, Weaver et al. crafted a survey study, utilizing a 40-question questionnaire to assign scores measured on three validated scales (PedsQLTM 4.0 Generic Core Parent Report for Toddlers Scales or the PedsQLTM Infant Scale, PedsQLTM 2.0 Family Impact Module, and the Functional Status Scale for Pediatric Hospitalization Outcomes). The authors concluded that in well-selected patients with Trisomy 18, an appreciable level of QOL may be maintained after morbid interventions such as cardiac surgery. 

Hepatoblastoma, a tumor of either epithelial or mesenchymal cells derived from the liver, is rarely observed in patients diagnosed with Trisomy 18 [11]. Our patient's case is unique as she underwent surgery and chemotherapy for multifocal hepatoblastoma, which is a rare complication of Trisomy 18 and has been reported in the literature infrequently [12]. Following definitive treatment, there is no evidence of disease relapse at 22 months post-surgery. The prognosis for hepatoblastoma is generally favorable with an overall survival of around 60-70% after treatment [13]. This underscores the importance of treating hepatoblastoma with curable intent as life expectancy lengthens with improving therapeutic options in patients with Trisomy 18. 

Our patient who has sought a second opinion and ultimately received non-regional medical care including multiple cardiac surgeries and an oncologic resection followed by adjuvant cytotoxic chemotherapy for a hepatoblastoma remains resilient and robust in her health and has remained out of the hospital for the past 18 months. Thanks to these life-saving measures, our patient now belongs to the 30% of survivors living with Trisomy 18 in the contemporary era. This has provided her family with additional time to cherish moments with their child. 

## Conclusions

Historically, Trisomy 18 has carried a grim prenatal prognosis, often resulting in the immediate perinatal loss of a newborn. However, in the contemporary era, seeking care at prominent tertiary academic centers staffed with expert multidisciplinary teams, including physicians, physical therapists, and social workers, provides life-saving possibilities that extend the lifespan of the infant while preserving the QOL for both the child and the family. As demonstrated in this case, patient outcomes for infants with Trisomy 18 are continually advancing and improving, reshaping the previously bleak prognosis associated with this congenital disorder.
